# Correlation Between Smell Recovery and Nasal Polyp Score in Patients Treated with Dupilumab: A Real-Life Retrospective, Observational, Monocentric Study

**DOI:** 10.3390/jpm15050164

**Published:** 2025-04-24

**Authors:** Giovanna Stilo, Giuliano Messina, Carmelo Lo Faro, Sara Ruta, Silvia Frangipane, Diana Mariut, Giorgia Giunta, Maria Elvira Distefano, Andrea Guglielmo Zappalà, Antonino Maniaci, Alberto Bianchi, Ignazio La Mantia

**Affiliations:** 1 Department of Medical and Surgical Sciences and Advanced Technologies “GF Ingrassia”, ENT Section, University of Catania, Via Santa Sofia, 95100 Catania, Italy; giuliano.messina1@studium.unict.it (G.M.); sararuta4@gmail.com (S.R.); uni383422@studium.unict.it (S.F.); dianacatalina.mariut@studium.unict.it (D.M.); giorgia.giunta@studium.unict.it (G.G.); maryelvira@oulook.it (M.E.D.); zappala.andrea@studium.unict.it (A.G.Z.); alberto.bianchi@unict.it (A.B.); i.lamantia@unict.it (I.L.M.); 2 Department of General Surgery and Surgical-Medical Specialties, Section of Maxillo Facial Surgery of Polyclinic “G. Rodolico—San Marco” University Hospital, University of Catania, 95123 Catania, Italy; carmelolofaro28@gmail.com; 3 Department of Medicine and Surgery, University of Enna Kore, 94100 Enna, Italy; antonino.maniaci@unikore.it

**Keywords:** CRSwNP, Dupilumab, biological therapy, olfactory disorders, type 2 inflammation

## Abstract

**Background**: Olfactory recovery plays an important role in improving the quality of life in patients with chronic rhinosinusitis with nasal polyposis (CRSwNP), and Dupilumab therapy shows promising results. The Nasal Polyp Score (NPS), Visual Analog Scale (VAS) for olfactory disorders (ODs), and the Sniffin’ Sticks 16-item identification test (SS-I) are three of the main indices of efficacy in CRSwNP treatment. Although mechanical obstruction appears to be a major cause of olfactory disorders in this condition, the three indices can often vary with different trends. Aim: The aim is to assess whether there is a correlation between the sense of smell recovery and the volumetric reduction in polyps and to assess how the reduction in NPS affects the improvement in olfactory symptoms. **Methods**: An observational monocentric retrospective cohort study was conducted on a sample of 50 patients, enrolled in treatment with Dupilumab for 12 months at the ENT Unit of Rodolico Hospital. We investigated the relationship between NPS changes and olfactory recovery using the Sniffin’ Stick 16-item identification test and the VAS for ODs at baseline and follow-up endpoints (1-, 3-, 6-, 9-, and 12-month assessments). **Results**: During the follow-up, according to the data in the literature, the patients showed a faster improvement in terms of SS-I and VAS for ODs than expressed in terms of NPS variation. **Conclusions**: This study shows that, in patients treated with Dupilumab, there is no strong correlation between the reduction in NPS and the recovery of the sense of smell evaluated by an improvement in the SS-I and VAS for ODs in the 12 months of follow-up, suggesting that, in patients with CRSwNP, the improvement in olfactory symptoms following treatment with Dupilumab is mainly related to its anti-inflammatory effects and not to the reduction in mechanical obstruction caused by nasal polyps.

## 1. Introduction

Chronic rhinosinusitis (CRS) is a chronic inflammatory pathology of the nasal–sinus cavities, with a prevalence in the general population of between 5 and 12% [1]. CRS can be phenotypically associated or not with nasal polyposis. Approximately 30% of subjects with CRS have the form associated with nasal polyposis [2].

Chronic rhinosinusitis with nasal polyposis (CRSwNP) is a chronic inflammatory disease involving disabling symptoms, such as nasal congestion, rhinorrhea/postnasal drip, impaired sense of smell (OI), facial pain/pressure, the presence of nasal polyps on endoscopy, and nasal and/or sinus opacification on computed tomography (CT) [1]. CRSwNP is a complex, multifactorial disease that significantly affects the quality of life (QoL) of patients and is further worsened in patients with comorbidities such as asthma, respiratory disease exacerbated by nonsteroidal anti-inflammatory drugs (NSAID-ERDs), or a history of sinonasal surgery [2]. CRSwNP is burdened by a high rate of recurrence after surgery, with approximately 40% of patients with CRSwNP experiencing the recurrence of the disease 18 months after the last surgery [2].

In many cases, CRSwNP is characterized by the activation of type 2 inflammatory pathways, with a series of concatenated effects that lead to an increase in the concentration of eosinophils (systemic and/or local), IgE (systemic or even just local), and interleukins IL-4, IL-5, and IL-13 [3,4]. Type 2 inflammation is driven by both innate (ILC2) and adaptive (Th2 cell) compartments of the immune system. These cytokines are produced by group 2 innate lymphoid cells (ILC2s) and TH2 cells [3].

The etiopathogenetic discoveries relating to type 2 inflammation have represented a real revolution in the management of this pathology, so the pathology is no longer classified only on the basis of the phenotypic distinction but also on the basis of the type of inflammation underlying the disease.

With the help of this new knowledge on the pathophysiological mechanisms linked to the type 2 inflammation underlying the pathology, we have experienced a change in the approach to the management of CRSwNP, mainly related to the new therapeutic opportunities represented by biological drugs [4].

CRSwNP compromises the QoL of affected patients significantly from a physical, psychological, and social point of view. Nasal obstruction, rhinorrhea, and headache create significant discomfort in the daily lives of these patients, compromising their work and emotional spheres. However, the most disabling symptom for patients is the decrease in or absence of smell, often accompanied by the loss of taste.

OI is the most annoying and disabling symptom in patients with CRSwNP, and above all, it is the symptom that most impacts their QoL [1]. Olfactory disorders have important psychological implications, with the appearance of depression and anxiety. The sense of smell is associated with memory and recollection, representing an important social sense in interpersonal relationships. Furthermore, the loss of smell represents a potential danger for those affected, as it makes it impossible to recognize spoiled food or detect the presence of fumes or harmful gases.

Olfactory disorders, in these patients, have a multifactorial etiology; the set of mechanisms linked to the edema of the neuroepithelium (neurosensory hypothesis) on the one hand and changes in the air flow inside the olfactory fissure (conductive hypothesis) [1] on the other contribute to this dysfunction.

The ideal treatment of CRSwNP should allow for the control of symptoms and improve QoL, along with an improvement in the endoscopic and radiological score. Encouraging results from clinical trials of biologics have been obtained, representing a possible new option in the treatment of uncontrolled CRS disease [1].

Dupilumab is a human monoclonal antibody derived from VelocImmune^®^ (Sanofi, Paris, France) that inhibits the interleukin (IL)-4 receptor α, the shared receptor component for IL-4 and IL-13, which are key cytokines involved in mediated synthesis 2 inflammation [5,6]. IL-4 and IL-13 are powerful mediators of type 2 inflammation, and both perform similar but also precise and peculiar functions. In particular, IL-4 is the main mediator of differentiation of Th2 lymphocytes and the development of B lymphocytes, while IL-13 is decisive in increased mucus secretion, hyper-reactivity of the airways and in tissue remodelling.

Dupilumab, with its anti-inflammatory action, can improve endoscopic scores linked to the size of polyps and QoL, and above all, it significantly improves olfactory symptoms.

The primary outcome was to evaluate the strong impact of Dupilumab on symptoms of olfactory impairment in subjects with severe CRSwNP [6,7]. The secondary objective was to evaluate whether the symptoms related to olfactory disability depended on the volume of nasal polyps or rather on the neuroinflammation that drives the pathology.

## 2. Materials and Methods

### 2.1. Study Design and Population

This is a retrospective, observational, real-life, monocentric cohort study. The study was approved as part of a large research program (prot. n 103/2021/PO) by the Human Medical Research and Ethics Committee of the University of Catania (approval code: 24121, date of approval 21 May 2021). This study was conducted following the Declaration of Helsinki. All participants provided written informed consent before data collection.

We enrolled 50 adult patients (≥18 years) with severe, uncontrolled chronic rhinosinusitis with nasal polyposis (CRSwNP) treated with Dupilumab for 12 months. Participants were assessed at baseline and follow-up endpoints (i.e., 1, 3, 6, 9, and 12 months). CRSwNP was diagnosed clinically with nasal endoscopy and imaging findings according to criteria from the European Position Paper on Rhinosinusitis and Nasal Polyps (EPOS 2020) [8]. Inclusion criteria were as follows: endoscopic Nasal Polyp Score (NPS) between 5 and 8 (minimum 2 in 1 side of nasal cavity) and Visual Analog Scale (VAS) score for olfactory disorders (VAS-OD) ≥ 7. Patients had a type 2 inflammation profile based on blood eosinophil count and serum IgE levels.

### 2.2. Exclusion Criteria

Patients with any of the following factors were excluded:

- History of administration of other monoclonal antibodies.
- Congenital or acquired immunodeficiencies; autoimmune disorders; or systemic neurodegenerative conditions (e.g., Parkinson’s or Alzheimer’s disease).
- Prior head and neck radiation therapy or sinonasal malignancies.
- Other nasal–sinus pathologies (e.g., antrochoanal polyps, inverted papillomas, acute sinusitis).
- History of anosmia or hyposmia unrelated to CRSwNP.

### 2.3. Treatment Protocol

All patients were treated with Dupilumab (300 mg) administered subcutaneously every 14 days for 12 months. Immunotherapy was administered as adjunctive therapy, consisting of Mometasone Furoate nasal spray (100 mcg per nostril, twice daily) and daily nasal saline irrigation. None of the patients required oral corticosteroid (OCS) cycles throughout the study period.

### 2.4. Outcome Assessments

Patients were assessed longitudinally at baseline (T0) and at subsequent follow-up timepoints: 1 month (T1), 3 months (T2), 6 months (T3), 9 months (T4), and 12 months (T5).

Individuals were evaluated by nasal endoscopy, with 0-degree rigid optics (Storz, 2.7 mm), with NPS ranging from 0 to 8 as the sum of the two nasal cavities.

A Sniffin’ Sticks 16-item identification test (SS-I) was used to assess olfactory function. It consists of a forced choice test with 16 odour pens, validated for clinical practice [9]. The scores range from 0 (no identification) to 16 (full identification). A Visual Analog Scale for olfactory disorders (VAS-OD) was also administered: a 0–10 scale whereby 10 means the most severe olfactory dysfunction. We further evaluated quality of life scores and symptom burden using the 22-item Sinonasal Outcome Test (SNOT-22).

### 2.5. Statistical Analysis

Statistical analyses for longitudinal datasets were employed for the analysis of data, considering the use of repeated measurements in individuals. The data were analyzed using R software (4.4.2 version), and statistical significance was considered with a *p*-value < 0.05. The continuous variables were summarized as mean ± standard deviation (SD) or median with interquartile range (IQR) and tested using Mann–Whitney or t-test, depending on the distribution of data. Variable categories were reported as n (percent).

Changes in scores (NPS, SS-I, VAS-OD, and SNOT-22) over time were assessed using linear mixed-effects models, accounting for within-subject correlations and inter-individual variability. Time was modelled as a fixed effect, and random intercepts were included to allow for patient-specific effects. Pearson or Spearman correlation coefficients (based on normality) were calculated to determine the relationship between NPS, SS-I, VAS-OD, and SNOT-22 scores at each timepoint.

We generated scatterplot matrices along with regression lines to visualize inter-variable correlations. All significant correlations also reported confidence intervals (CIs) and effect sizes.

Individual patient trajectories for SS-I and VAS-OD were plotted over time to visualize variability in response to the treatment.

Individual slopes of SS-I improvement were calculated and used to examine their relationship with NPS reduction using scatterplots.

Subgroup analysis was performed to detect potential confounders (i.e., asthma, NSAID-exacerbated respiratory disease, NSAID-ERD, or allergic features). Independent t-tests or Mann–Whitney U tests were used to compare olfactory recovery and NPS reductions between subgroups.

## 3. Results

Overall, 50 patients (21 females) completed the follow-up; the mean age was 53.04 years. All patients had CRSwNP and were being treated with Dupilumab. Complete baseline characteristics of the study population are listed in Table 1. Thirty-seven patients (70%) had asthma as a comorbidity, and thirty-nine patients (74%) had a positive history of inhalant allergy diagnosed by the Prick Test. Eleven patients (21%) demonstrated nonsteroidal anti-inflammatory drug (NSAID)-exacerbated respiratory disease (N-ERD). None had atopic dermatitis.

Complete baseline characteristics of the study population are listed in Table 1.

All patients performed therapy with Dupilumab 300 mg, by subcutaneous injection every 14 days. At the same time, the patients underwent local therapy with nasal spray based on Mometasone Furoate 100 mcg; the daily dosage was one puff per nostril twice a day every day. No patient needed OCS cycles. All patients concluded the observation period after 48 weeks, and all patients tolerated Dupilumab therapy well. We had three cases of hypereosinophilia (>3 × 109/L) three months after the start of treatment, without organ repercussions and with no symptoms perceived by the patient. Hypereosinophilia was treated with a course of deflazacort 30 mg orally, one tablet/day for 7 days, with resolution of hypereosinophilia (<1.5 × 109/L).

**Figure 1:**
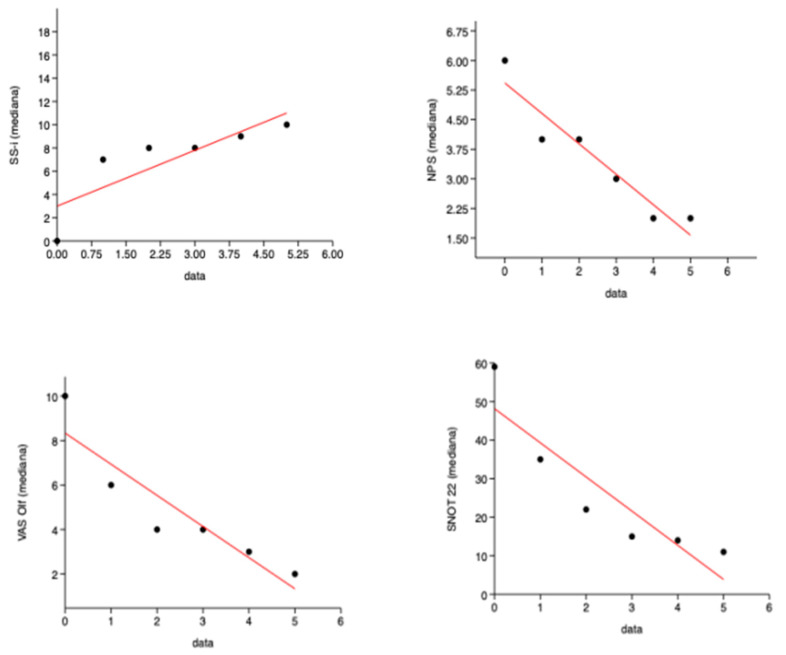
Results. The mean total score of the Nasal Polyp Score (NPS), Sniffin’ Sticks 16-item identification test (SS-I), Visual Analog Scale (VAS) for olfactory disorders (OD), and the 22-item Sinonasal Outcome Test (SNOT-22), at T0, T3, T6, T9, and T12.

The SS-I demonstrated a statistically significant (*p* = 0.037) improvement in OI (Figure 1).

**Figure 2:**
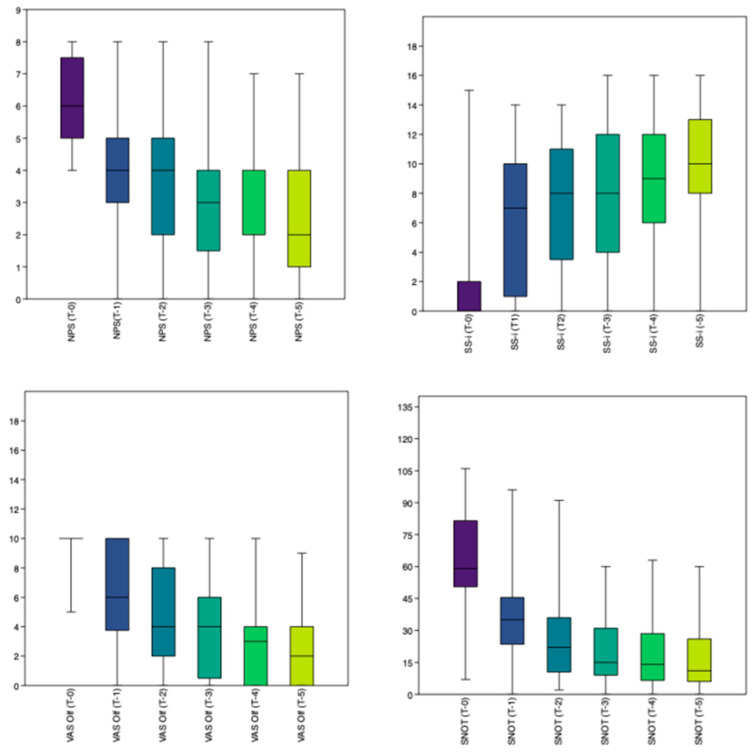
A low negative correlation was found between NPS-T0 and SS-I T0 (ρ = −0.35; r2 = 0.046; *p* = 0.013), as well as in all subsequent follow-ups.

The improvement in smell did not demonstrate a correlation with NPS, so the two variables were independent (Figure 2).

### Linear Mixed Model

Our linear mixed model reported a substantial proportion of variance in the outcome measures: NPS (0.697), SNOT-22 (0.752), SS-I (0.602), and VAS Smell (0.626). The marginal R^2^ values, expressed by the fixed effects alone, were lower. A likelihood ratio test (LRT) confirmed significant differences in all models (*p* < 0.001) (Table 2).

The fixed effect of time was significant for all variables, with F-statistics indicating strong evidence of temporal improvement (NPS: F(5257) = 65.8; SNOT-22: F(5257) = 109; SS-I: F(5257) = 42.0; VAS Smell: F(5257) = 71.6). Random effects showed significant interindividual variability. In addition, post hoc pairwise comparisons revealed significant differences between baseline (T0) and the 12-month follow-up (T12) for all outcome measures. Specifically, NPS showed a mean reduction of 3.72 points (*p* < 0.001), while SNOT-22 demonstrated a substantial decrease of 47.8 points (*p* < 0.001). Similarly, SS-I scores improved significantly by 7.79 points (*p* < 0.001), and VAS Smell showed an increase of 7.25 points (*p* < 0.001), confirming a clinically meaningful improvement in olfactory function.

**Figure 3:**
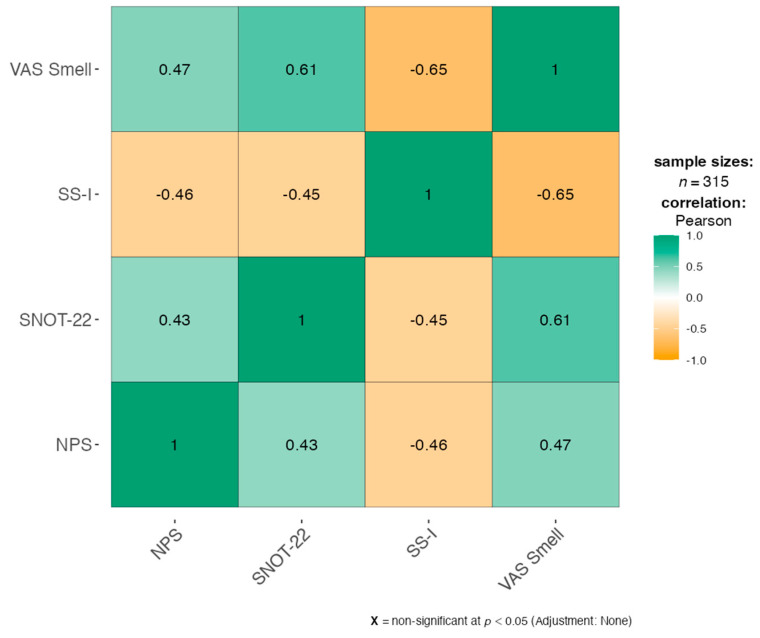
Correlation Matrix among dependent variables.

Correlation analysis showed that the NPS was positively correlated with the SNOT-22 (r = 0.43) and VAS Smell (r = 0.47), suggesting that higher polyp burden is associated with more severe sinonasal symptoms and olfactory impairment (Figure 3).

Comparatively, SS-I scores were negatively correlated with both NPS (r = −0.46) and VAS Smell (r = −0.65), further supporting the finding that greater nasal polyp volume and subjective odour dysfunction are associated with diminished objective olfactory scores. The above findings indicate that the olfactory dysfunction in CRSwNP could be more driven by inflammatory mechanisms than mechanical blockage.

In the multi-linear regression, the complex association between sinonasal symptom burden and olfactory dysfunction in patients with CRSwNP was highlighted. The strong positive correlation between VAS Smell and both SNOT-22 (β = 0.0759, *p* < 0.001) and NPS (β = 0.4278, *p* < 0.001) noted a correlation between worsening symptom severity and polyp burden and increased subjective impairment of smell (Table 3).

In contrast, the SNOT-22 scores showed a negative correlation with the SS-I scores (β = −0.0612, *p* < 0.001), and the NPS (β = −0.7208, *p* < 0.001) emphasized a relationship between symptom burden and polyp size with objectively poorer olfactory identification. The SS-I model (R^2^ = 0.288) explained lower variance than the VAS Smell model (R^2^ = 0.430). Intriguingly, sex was not a significant predictor in both models (VAS Smell: β = 0.1041, *p* = 0.745; SS-I: β = −0.4844, *p* = 0.305).

## 4. Discussion

Although often underestimated in daily life, some clinical studies have demonstrated in patients with olfactory dysfunctions the onset of mood disorders, depression, eating disorders, as well as difficulties in interpersonal relationships. Katotomichelakis et al. showed that the quality of life and mental health of patients with chronic sinus diseases have a direct positive relationship with improved sense of smell [10]. CRSwNP also causes sleep disturbances, linked to nasal obstruction caused by nasal polyps, which contribute to the deterioration of the mood of these patients, developing cognitive–behavioural changes and mood disorders [11]. Loss of or reduction in sense of smell is one of the most troublesome and difficult-to-treat symptoms in CRSwNP [12].

The sense of smell is involved in a series of social behaviours linked to aggression, reproduction, recognition of friends and enemies, and memory, which make it a “social sense” in all respects [13]. It is therefore easy to understand how an impairment of smell is often perceived by the patient with CRSwNP as the most disabling symptom. 

Olfactory recovery is often the first objective set in the treatment of chronic rhinosinusitis with nasal polyposis, precisely because of its high correlation with quality of life.

Although enormous efforts have been made to fully understand the pathogenetic mechanisms associated with the damage of type 2 inflammation in chronic rhinosinusitis with nasal polyposis, there are some pathogenetic aspects such as the origin of the damage to the olfactory mucosa and whether the trigger is represented exclusively by neuro-inflammation. In this study, we tried to better understand the clinical impact of treatment with biological drugs, and specifically with Dupilumab, on the olfactory mucosa. The improvement of olfactory function is a point of crucial importance in the evaluation of patients with CRSwNP. For this reason, olfactory recovery was included in the selection criteria for subjects who are suitable for treatment with a biological drug and was also included as a response parameter to treatment with monoclonal antibodies in the European Forum for Allergies and Airway Diseases (EUPHOREA) and in EPOS [14]. According to recent research, the difficulty in recovering from OI with classic therapy is an indicator of the severity of the pathology. For this reason, it is necessary to carefully evaluate this symptom and its changes. From the above, it is clear how fundamental it is for ENT specialists to be familiar with the most common identification tools for olfactory disorders in clinical practice [15]. In the present study, we reported the real-life clinical outcomes of patients affected by CRSwNP treated with Dupilumab at a tertiary referral centre.

We noticed at baseline an important impact of CRSwNP on the patient’s quality of life; in particular, the olfactory impairment was the most disabling symptom, demonstrated by the SNOT-22 and the VAS for OD [11]. The latter had a value > 6 in all our patients. In the subsequent evaluations at 1 and 3 months, a significant improvement in olfactory impairment was noted, highlighted by the results of the SNOT.22 and the VAS for OD, which presented a value <4 in all our patients. Despite a significant improvement in OD, this evidence did not correlate with a decrease in the volume of nasal polyps on the NPS [14].

SNOT-22 was not correlated with SS-I, but we found a correspondence between three items of SNOT-22. Specifically, the items “decreased sense of smell/taste”, “sadness”, and “frustration/restlessness/irritability” correlated with SS-I.

The correlation between the last two items (“sadness” and “frustration/restlessness/irritability”) and SS-I strengthens the evidence of the strong impact of OD on QoL, underlining how much the “hypo-anosmia” symptom in patients with CRSwNP is the most disabling for the patient [13].

Therefore, the improvement of olfactory VAS and SS-I did not correlate with NPS. This demonstrates the fact that olfactory impairment, which represents one of the key symptoms of CRSwNP, does not depend on the volume of nasal polyps, but rather is strictly dependent on the pathogenetic mechanisms linked to type 2 inflammation, which determine the pathology [14].

It is possible to hypothesize that, at the basis of the OD in CRSwNP and the olfactory improvement following treatment with Dupilumab, there are immunological mechanisms with direct or indirect involvement of interleukins (ILs) and, in particular, IL-4, IL-5, IL- 13, and eosin-ophiles [14]. The olfactory neuroepithelium contains three important cell types of the peripheral sensory system, including olfactory sensory neurons (OSNs) [15], which are very sensitive to mediators of immunophlogosis found in CRSwNP [15]. The infiltration of common mediators of type 2 inflammation into the olfactory neuroepithelium could be the cause of the dysfunction of the peripheral olfactory system. Consequently, inflammation of the epithelium could explain the olfactory disorder through its neuroinflammatory genesis [15].

Eosinophilic infiltration could play a fundamental role in the onset of olfactory loss.

Furthermore, increased levels of inflammatory cytokines in the olfactory cleft have been correlated with OD; elevated levels of IL-4, IL-5, and IL-13 are indeed associated with worse scores in olfactory identification tests; smell disorders have a direct correspondence with the elevated levels of cytokines IL-4 and IL-13, although the key role of the IL-4 and IL-13 pathways and their possible regulatory implications on the neurogenesis and homeostasis of olfactory neurons has not yet been fully clarified and explained [16].

However, our model reinforced the complex link between sinonasal symptom burden and olfactory dysfunction in patients with CRSwNP. The strong positive correlation between SNOT-22 and VAS Smell (β = 0.0759, *p* < 0.001) and NPS (β = 0.4278, *p* < 0.001) demonstrated that increased subjective olfactory impairment correlates with more severe clinical symptoms and a greater polyp burden. It indicates that patients with more severe sinonasal symptoms perceive their sense of smell as more impaired, supporting the involvement of inflammatory processes in olfactory dysfunction.

In contrast, the negative effects of SS-I scores on both SNOT-22 (β = −0.0612, *p* < 0.001) and self-reported NPS (β = −0.7208, *p* < 0.001) reinforced the correlation between increased symptom burden and polyp size with objectively poorer olfactory identification. Although mechanical obstruction may contribute to functional deficits, these results suggest that inflammatory-induced changes within the olfactory neuroepithelium are a predominant driver of reduced olfactory function. The lower amount of variance explained by the SS-I model (R^2^ = 0.288) compared to the VAS Smell model (R^2^ = 0.430) hints that subjective perception of olfactory function may be affected by at least some additional factors not covered in this study.

We found that sex itself was not a significant predictor in either model (VAS Smell: β = 0.1041, *p* = 0.745; SS-I: β = −0.4844, *p* = 0.305), suggesting that biological sex differences do not significantly impact olfactory function impairment in CRSwNP. This agrees with the previous literature that supports the idea that although sex-related differences in olfactory processing may exist, inflammation and nasal polyp burden are the main determinants of olfactory dysfunction in CRSwNP. Although surgery may be beneficial in partially restoring olfactory function, olfactory dysfunction outcomes after surgery are not linear in CRSwNP patients, and the improvement in OD over time among eosinophilic CRSwNP patients appears not to be well maintained after surgery [17]. The goal is to identify a personalized therapeutic path for each patient. Biological therapy represents a revolution in the treatment of nasal polyposis capable of modifying the quality of life of our patients; therefore, it is essential for an appropriate prescription and a correct classification of the patient at baseline, which is also guaranteed by a multidisciplinary approach to the pathology. The specialist must initiate a multidisciplinary study and management process, which involves an allergist, pulmonologist, radiologist, and immunologist, to evaluate the presence of comorbidities such as allergy and asthma, study the immunological characteristics of the disease, and offer the patient medical treatment that is as personalized as possible. We must increasingly apply precision medicine, which will allow us to identify the correct treatment for the correct patient.

Endoscopic sinus surgery (ESS) for medically refractory CRS is performed primarily to promote good sinus function and to enhance the effect of intranasal corticosteroid treatment. However, olfactory changes after ESS depend on the number of previous surgeries and are not predictable [18,19]. Although olfactory dysfunction in chronic rhinosinusitis is expected to evolve to restitution ad integrum after surgical treatment, with endoscopic sinus surgery and long-term local medical therapy, a large number of patients still experience permanent olfactory alteration.

## 5. Conclusions

Biological drugs are among “molecular target” therapies and represent the future of medicine. Their mechanism of action makes them capable of binding specifically to a molecular “target” (receptor, enzyme, growth factor, etc.), and for this reason, they are compared to intelligent bullets capable of inhibiting, as in the case of chronic rhinosinusitis with nasal polyposis, specific mediators of inflammation. The advantage of these therapies is that being able to offer our patients a targeted, tailored, and effective therapy capable of improving the quality of life of our patients, nasal obstruction, and olfactory and disease-related symptoms reduces the need for new surgeries and taking high amounts of systemic corticosteroids. The goal is to achieve the personalization of care. In agreement with other data in the literature, a statistically significant correlation was not found between NPS and SS-I values [1]. It is therefore conceivable that the mechanisms underlying olfactory recovery after Dupilumab therapy are, partially or fully, independent of the volumetric reduction in the polyps and that they could depend exclusively on the resolution of the local inflammation exerted by the biological drug. In conclusion, our analysis demonstrated that Dupilumab is a monoclonal antibody capable of improving olfactory function and acting rapidly in this regard [14]. The olfactory improvement appears to be linked to the anti-inflammatory action of the biological therapy, rather than to the size and volume of the nasal polyps. The mechanism of action of Dupilumab, linked to the inhibition of signal transduction of IL-4 and IL-13 [12], makes it highly effective in the treatment of pathologies related to type 2 inflammation and would appear to be the cause of the olfactory improvement in these patients. Our study has certainly different limitations including retrospective design, the monocentric setting, and small sample size, and future studies may be necessary to clarify and confirm our results on this topic.

## Figures and Tables

**Table 1:** Patient characteristics at baseline.

.	Average	Min/Max	Median
Age (years)	53.04	26–87	52
Gender (male; female)	M 29 (58%)	F 21 (42%)	-
Height (cm)	169	150–190	170
Weight (Kg)	73,7	49.99	74
BMI	25.68	17.1–38	25.3
Smokers	-	8(15%)	-
NSAID.ERD	-	11(21%)	-
Allergy	-	39(74%)	-
Asthma	-	37(70%)	-
>2 Surgery	-	15(28%)	-

**Table 2:** Linear mixed model and post hoc comparison.

**Linear Mixed Model**															
**Variable**	**Conditional R2R^2R2**		**Marginal R2R^2R2**	**LRT χ2\chi^2χ2**		**Fixed Effect (Time)**	**F (df, res df)**		***p*-Value**		**Random Effect Variance (Intercept, PatientID)**		**Residual Variance**		**ICC**
NPS	0.697		0.317	265.347		Time	65.8 (5, 257)		<0.001		1.90 (SD = 1.38)		1.51 (SD = 1.23)		0.556
SNOT-22	0.752		0.433	327.543		Time	109 (5, 257)		<0.001		198 (SD = 14.1)		154 (SD = 12.4)		0.562
SS-I	0.602		0.267	195.171		Time	42.0 (5, 257)		<0.001		8.25 (SD = 2.87)		9.80 (SD = 3.13)		0.457
VAS Smell	0.626		0.427	235.910		Time	71.6 (5, 257)		<0.001		2.80 (SD = 1.67)		5.27 (SD = 2.29)		0.347
**Fixed Effect Estimates**															
**Variable**		**Time Comparison**			**Estimate**			**SE**		**95% CI (Lower–Upper)**		**t-value**		***p*-value**	
NPS		0 vs. 12			−3.72			0.239		−4.19 to −3.25		−15.56		<0.001	
SNOT-22		0 vs. 12			−47.8			2.41		−52.6 to −43.1		−19.9		<0.001	
SS-I		0 vs. 12			7.79			0.608		6.60 to 8.99		12.82		<0.001	
VAS Smell		0 vs. 12			−7.25			0.446		−8.12 to −6.37		−16.25		<0.001	
**Post Hoc Comparison**															
**Variable**		**Time Comparison**			**Mean Difference**			**SE**		**t-value**		**df**		***p*-value**	
NPS		0 vs. 12			3.72			0.239		15.56		257		<0.001	
SNOT-22		0 vs. 12			47.85			2.41		19.87		257		<0.001	
SS-I		0 vs. 12			−7.79			0.608		−12.82		257		<0.001	
VAS Smell		0 vs. 12			7.25			0.446		16.25		257		<0.001	

**Table 3:** Multiple linear models.

Model Coefficients—VAS Smell					Model Coefficients—SS-I				
Predictor	Estimate	SE	t	*p*	Predictor	Estimate	SE	t	*p*
Intercept	0.9502	0.36226	2.623	0.009	Intercept	116.622	0.5336	21.86	<0.001
SNOT-22	0.0759	0.00715	10.606	<0.001	SNOT-22	−0.0612	0.0105	−5.81	<0.001
NPS	0.4278	0.07964	5.372	<0.001	NPS	−0.7208	0.1173	−6.14	<0.001
Sex: F–M	0.1041	0.31977	0.326	0.745	Sex: F–M	−0.4844	0.4710	−1.03	0.305
Model	R	R^2^			Model	R	R^2^		
1	0.655	0.430			1	0.537	0.288		

## Data Availability

The original contributions presented in this study are included in the article. Further inquiries can be directed to the corresponding author.
